# EAMA: Empirically adjusted meta-analysis for large-scale simultaneous hypothesis testing in genomic experiments

**DOI:** 10.1371/journal.pone.0187287

**Published:** 2017-10-31

**Authors:** Sinjini Sikdar, Somnath Datta, Susmita Datta

**Affiliations:** Department of Biostatistics, University of Florida, Gainesville, Florida, United States of America; University Hospital Jena, GERMANY

## Abstract

Recent developments in high throughput genomic assays have opened up the possibility of testing hundreds and thousands of genes simultaneously. However, adhering to the regular statistical assumptions regarding the null distributions of test statistics in such large-scale multiple testing frameworks has the potential of leading to incorrect significance testing results and biased inference. This problem gets worse when one combines results from different independent genomic experiments with a possibility of ending up with gross false discoveries of significant genes. In this article, we develop a meta-analysis method of combining p-values from different independent experiments involving large-scale multiple testing frameworks, through empirical adjustments of the individual test statistics and p-values. Even though, it is based on various existing ideas, this specific combination is novel and potentially useful. Through simulation studies and real genomic datasets we show that our method outperforms the standard meta-analysis approach of significance testing in terms of accurately identifying the truly significant set of genes.

## Introduction

In genomic experiments and association studies, meta-analysis is a popular tool for pooling results from multiple experiments and studies to reach an overall decision. In recent times, rapid progress in technology has led to major development of high throughput genomic assays. This means that hundreds and thousands of genes are now being analyzed at the same time. Thus, the level of simultaneous inference has undergone a huge surge over the last decade. Development of novel meta-analysis approaches is crucial for such settings since the sample size of individual experiments are generally small compared to the number of genes leading to low power of statistical detection from them after adjusting for multiplicities. However, there has not been much change in the meta-analysis methods to accommodate this large-scale aspect of the underlying inference and the possibility of underlying hidden factors that act as confounders. For example, in testing for the significance of genes in disease studies, more or less the same meta-analysis methods are being applied to experiments involving hundreds and thousands of genes as were initially developed for experiments involving a few number of candidate genes. One common practice is to use Fisher’s method [1] for combining the p-values from different testing problems involving the same overall hypothesis. The individual p-values are calculated for one gene at a time and the negative log-transformed p-values are combined to form a chi-squared test statistic under the assumption that they are individually uniformly distributed under the null hypothesis. However, as pointed out by Efron [2], in large-scale multiple testing problems, the “marginal” or “empirical” distribution of the p-values may not be uniformly distributed. Consequently, the distributional assumption of Fisher’s combined test statistic becomes questionable. To draw “better” inference, adjustments are needed to ensure that all the p-values from individual experiments are uniformly distributed so that the Fisher’s method of combining the individual p-values can be properly implemented.

In single hypothesis test framework, the main aim is to reject the null hypothesis in favor of some “interesting” alternative hypothesis with high power, say 80%. But in large-scale multiple testing framework involving, say, 10,000 hypotheses related to 10,000 genes, rejection of 80% of 10,000, i.e. 8,000, null hypotheses is no longer a desired outcome. Rather, the aim of such large-scale testing framework is to identify a small set of “interesting” cases or genes, usually less than 10%, which can be pursued for further investigation. The advantage of having large number, e.g., 100 or more, of hypotheses over a single hypothesis is that it enables the estimation of empirical null distribution avoiding the dependency on theoretical asymptotic null distribution, as pointed out by Efron [2]. The use of this empirical null is more appropriate for addressing the goal of large-scale hypotheses testing problems. This is particularly relevant in large observational studies which are often characterized by the presence of unobserved variable effects (e.g., batch effects) or unmeasured/missed confounding factors. Unlike the theoretical null, the empirical null distribution, automatically, takes into account the effects of the additional variation (and also small to moderate biases). This can have an even more serious consequence in meta-analyses of large scale genomic experiments where a number of potentially low-powered study results are combined in order to achieve significance. A motivating example can be found in [3] where the authors have discussed the fact that not adjusting for the potential confounder effects can lead to poor results of meta-analysis as shown in their genomic meta-analysis of a number of studies involving major depressive disorder. However, a p-value combination method such as Fisher’s method [1] may lead to incorrect findings if at least one of such study contains hidden sources of variation leading to a violation of the theoretical null distributional assumption for that component study. A relevant example of such inferences based on Fisher’s method can be found in the meta-analysis of lung cancer data in a later section of this article.

In particular, a possible consequence of combining unadjusted or incorrectly adjusted p-values through Fisher’s method [1] in a large-scale multiple testing situation is the prevalence of large number of false discoveries when some underlying hidden variable plays the role of a confounder. Occurrences of false discoveries are common in large scale DNA microarray experiments where the aim is to detect genes that are differentially expressed between two or more biological conditions. A good example in this context can be the well-known study of [4] which aimed to identify the differentially expressed genes between two types of genetic mutation of breast cancer, namely, “BRCA1” and “BRCA2” mutations. In this study 3266 genes were analyzed, out of which 51 genes turned out to be significant initially at the p-value cut-off of 0.001. Later on it was shown that the chosen cut-off is expected to produce substantial false positives, and the authors had to lower the cut-off resulting in the lower number of significant genes. Details of this study results can be found in [5]. This highlights the possibility of inaccurate scientific conclusions due to the occurrences of false discoveries in large-scale experiments. A possible remedy to this problem is to make adjustments using the false discovery rate (FDR), developed by [6], which have been widely used in methods for analyzing data from genomic experiments [7–10]. However, using the aforementioned breast cancer microarray data, Efron [2] showed that even adjusting for FDR may not be enough to restrict false discoveries if the underlying assumption of the standard normal distribution of the test statistic is under suspicion. Instead, he advocated using the “empirical null” in order to make the calls while using a local false discovery rate calculation. Motivated by this, we develop a meta-analysis method called Empirically Adjusted Meta-analysis (EAMA) that does not combine the raw p-values–they are first transformed, where the amount of transformation depends on the discrepancy between the empirical and the theoretical null (e.g., uniform distribution in case of p-values), before they are Fisher-combined. Of course, a multiple hypothesis method such as Benjamini–Hochberg (1995) [6] is applied at the end to make the significance calls. We show that this procedure is very effective in reducing the FDR and increasing specificity in a variety of situations which are affected by the presence of some hidden confounders.

## Experimental framework

### Simulation studies

In order to evaluate the performance of our proposed method (EAMA), described in the Methods section, for accurate identification of significant genes, we simulated datasets mimicking multiple genomic experiments. We simulated continuous expression datasets as obtained from microarray experiments, as well as, count datasets which are found in next generation sequencing experiments. Besides, we also considered the possible presence of some unknown hidden variables or confounders that often impact the results of the genomic experiments. It is our interest to check the performance of EAMA in circumstances affected by the presence of hidden variables or confounders. Details of the data generation methods are described below.

#### Generation of continuous data (microarray based gene expression)

We generated a number of simulation studies involving multiple genomic experiments. We had datasets obtained from *M* = 10 independently generated experiments, where each experiment had data on *G* = 1000 genes and *N* = 20 subjects distributed equally over two groups (for example, case and control). The first 10 subjects were considered to be in one group and the remaining 10 subjects in the other. The (log) expression levels of *G* genes for a typical experiment *m* were generated as follows.

Let *Y*_*ijk*_ be the (log) expression value corresponding to the *i*^*th*^ gene belonging to the *k*^*th*^ subject and *j*^*th*^ group. The (log) expression values (*Y*_*ijk*_) of genes were generated using a linear model as given below:

$$Y_{ijk}=\mu +G_{i}+V_{j}+{\left(GV\right)}_{ij}+W_{ijk}+e_{ijk}$$ (1)

where *μ* denotes the general mean effect in the model, *G*_*i*_ is the effect of the *i*^*th*^ gene, *V*_*j*_ is the effect of the *j*^*th*^ group, (*GV*)_*ij*_ is the interaction effect of the *i*^*th*^ gene and the *j*^*th*^ group, *W*_*ijk*_ is the effect of a latent confounder on the *i*^*th*^ gene of the *k*^*th*^ subject in the *j*^*th*^ group, *e*_*ijk*_ is the error component corresponding to the *i*^*th*^ gene of the *k*^*th*^ subject in the *j*^*th*^ group.

Here,

$$i=1,2,\ldots ,\;G;\;j=1,2;\;k=\left\{\begin{array}{cc} 1,\ldots ,10 & for\;j=1\\ 11,\ldots ,\;N & for\;j=2 \end{array}\right.$$

For each of the *M* independent experiments, the gene (log) expression profiles were generated as mentioned above. For our simulation studies on microarray data, the following two simulation settings were considered.

**Setting 1**: In this setting, we simulated the microarray datasets for each experiment using (1) assuming that there was no effect of any hidden confounder in the model. To achieve this, we set *W*_*ijk*_ = 0 for 1 ≤ *i* ≤ *G*; *j* = 1,2; *k* = 1,2,…, *N* in (1).

For simplicity, we further assumed that all the main effect terms were zero. That is, we set *μ* = 0, *G*_*i*_ = 0 for 1 ≤ *i* ≤ *G* and *V*_*j*_ = 0 for *j* = 1,2 in (1). The *e*_*ijk*_*s* denote mean zero random errors. We generated these random errors *e*_*ijk*_, in (1), independently from N(0, 0.8^2^) distribution under the assumption that all the genes in the datasets were independent.

In our simulations, we set 70 genes as differentially expressed between the two groups of subjects. In particular, we considered the differences in magnitudes of differential (log) expressions of these 70 genes between the two groups to be 8. For this, the interaction effects between the genes and the groups were generated as given below:

For 1 ≤ *i* ≤ 20, (*GV*)_*i*1_ = −4, (*GV*)_*i*2_ = 4
For 21 ≤ *i* ≤ 70, (*GV*)_*i*1_ = 4, (*GV*)_*i*2_ = −4
For 71 ≤ *i* ≤ *G*, (*GV*)_*i*1_ = (*GV*)_*i*2_ = 0

This data generation was repeated for each of the *M* independent experiments.

**Setting 2**: In this setting, we wanted to evaluate the performance of EAMA in the presence of hidden confounder in the model. The effects of the latent variable (*W*) in (1) were generated in this setting in such a way that it varied not only over the two groups of subjects and different groups of genes but also over different experiments. So, here we generated *W*_*ijk*_ as *W*_*ijk*_ = *u*_*ijk*_*I*(*s*_*ijk*_ = 1), where *s*_*ijk*_ ~ Bernoulli(0.4). When *s*_*ijk*_ = 0, *W*_*ijk*_ = 0, which implied that there was no effect of the latent confounder on the *i*^*th*^ gene of the *k*^*th*^ subject in the *j*^*th*^ group. On the other hand, when *s*_*ijk*_ = 1, *W*_*ijk*_ = *u*_*ijk*_, i.e., the effect of the latent confounder was given by *u*_*ijk*_ for the *i*^*th*^ gene of the *k*^*th*^ subject in the *j*^*th*^ group. Here, *u*_*ijk*_ was generated depending on the gene, subject group, and experiment ID (*m*) as follows:

$$u_{i1k}\sim \left\{\begin{array}{cc} N(-1+m,\;0.01^{2}) & for\;1\leq i\leq 20;\;k=1,2,\ldots ,10\\ N(2+m,\;0.01^{2}) & \;\;for\;21\leq i\leq 70;\;k=1,2,\ldots ,10\\ N(5+m,\;0.01^{2}) & \;\;\;for\;71\leq i\leq G;\;k=1,2,\ldots ,10 \end{array}\right.$$

and

$$u_{i2k}\;\sim \;\left\{\begin{array}{cc} N(-1+\delta +m,\;0.01^{2}) & for\;1\leq i\leq 20;\;k=11,12,\ldots ,N\\ N(2+\delta +m,\;0.01^{2}) & \;\;for\;21\leq i\leq 70;\;k=11,12,\ldots ,N\\ N(5+\delta +m,\;0.01^{2}) & \;\;\;for\;71\leq i\leq G;\;k=11,12,\ldots ,N \end{array}\right.$$

Here, *δ* denotes the magnitude of the difference between the means of the distributions of *u*_*ijk*_ in the two groups. For our simulation scenarios, we considered *δ* = 4 unless mentioned otherwise. Generation of *W*_*ijk*_ using the above design represents a situation where the gene expression values depend on the group status of the subjects and also on some unobserved features of the experiment (for example, age and gender of the subjects, geographical location of the experiment, etc.) which are often present in observational studies. We generated *e*_*ijk*_*s*, in (1), independently from N(0, 0.05^2^) distribution. All the other variables in (1) were generated in the same way as in Setting 1 for each of the *M* independent experiments.

After generating the microarray datasets, the set of differentially expressed genes were identified for each of the *M* experiments using “limma” in Bioconductor [11] and the corresponding raw p-values of all the genes under study were stored. Note that while identifying the set of differentially expressed genes using “limma” we did not consider the effect of any unmeasured/hidden confounding factors that may be present in the simulation model. This is because, although affecting the outcome, these factors remain unaccounted in practice, the very reason that they are labelled as “hidden” or “latent”. We then applied our method EAMA to obtain the set of significant genes.

We also considered a simulation scenario where the magnitude of differential expression among the set of differentially expressed genes was reduced. Moreover, we simulated a scenario where we introduced an effect of a hidden variable which does not act as a confounder. The above-mentioned scenarios are described below.

#### i) Reduction in the difference in magnitude of the expression levels of the genes

We considered the situation where the magnitude of the difference in the (log) expression levels of the 70 differentially expressed genes between the two groups was reduced. This was reflected through the reduction of magnitudes of the interaction effects between genes and groups in (1). Here the interaction effects (*GV*) were generated as given below:

For 1 ≤ *i* ≤ 20, we set (*GV*)_*i*1_ = −2, (*GV*)_*i*2_ = 2
For 21 ≤ *i* ≤ 70, we set (*GV*)_*i*1_ = 2, (*GV*)_*i*2_ = −2
For 71 ≤ *i* ≤ *G*, we set (*GV*)_*i*1_ = (*GV*)_*i*2_ = 0

So, the difference in magnitude of the (log) expression levels of the differentially expressed genes between the two groups was 4 instead of 8 as considered in the previous scenario.

#### ii) Presence of a hidden variable which does not act as a confounder

In addition to Setting 1 and Setting 2, we also checked the situation where a hidden variable, although present and affects the outcome, does not act as a confounder. We refer to this setting as Setting 3. Here we considered a simulation scenario where the distribution of the latent variable (*W*) in (1) is the same in the case and the control groups of subjects i.e. the effect of the hidden variable on the outcome does not vary significantly between the two groups of subjects. Here, *W*_*ijk*_ = *u*_*ijk*_ *I*(*s*_*ijk*_ = 1), where *s*_*ijk*_ ~ Bernoulli(0.4) and *u*_*ijk*_ was generated for the *m*^*th*^ experiment as:

$$u_{ijk}\sim \left\{\begin{array}{cc} N(2+m,\;0.1^{2}) & for\;1\leq i\leq 20;\;k=1,2,\ldots ,N\\ N(2+m,\;0.1^{2}) & \;\;for\;21\leq i\leq 70;\;k=1,2,\ldots ,N\\ N(2+m,\;0.1^{2}) & \;\;\;for\;71\leq i\leq G;\;k=1,2,\ldots ,N \end{array};\;j=1,2\right.$$

The differences in magnitudes of differential (log) expressions of the 70 differentially expressed genes between the two groups were 2.

In addition to the above simulation scenarios, we also considered several other variations by introducing correlation among some of the genes under study, increasing the number of genes involved, and changing the number of independent experiments. Moreover, we considered a simulation scenario where the effect of the confounder exists in individual experiments, but the confounding effect tends to get nullified on combining all the experiments. Details of these aforementioned analyses can be found in the Supporting information (see S1 Text).

#### Generation of count data (NGS based gene expression)

We also generated realistic NGS-like datasets for our simulation experiments using a popular NGS-simulator called SimSeq [12]. SimSeq generates read counts in two treatment groups for a known set of differentially expressed genes based on a real RNA-sequencing dataset. Here, we generated a count data using SimSeq and kidney renal clear cell carcinoma data as the source dataset [13]. The kidney renal clear cell carcinoma dataset consisted of 20,531 genes and 144 paired samples with tumor and non-tumor replicate. We filtered the kidney renal clear cell carcinoma dataset by including only those genes which had more than one hundred non-zero read counts so that the simulated dataset did not include all zero read counts. From the reduced source dataset we generated read counts with *G* = 5000 genes and *N* = 60 subjects distributed equally over two groups. Out of these 5000 genes, 1000 genes were differentially expressed.

Similar to the previous simulated scenarios involving expression datasets, we also assumed that there existed an effect of a hidden confounder in the count dataset. In order to achieve this, we independently generated another set of read counts using SimSeq with 5000 genes and 60 subjects as before where 1000 genes were differentially expressed. For the *i*^*th*^ gene, *j*^*th*^ group and *k*^*th*^ subject we generated a random observation *s*_*ijk*_ from Bernoulli(0.4), *i* = 1,…,5000, *j* = 1,2, and *k* = 1,…,60. When *s*_*ijk*_ = 1, we added the two read counts and divided the resulting sum by two in order to maintain the original magnitude of the read counts, for the *i*^*th*^ gene in the *j*^*th*^ group for the *k*^*th*^ subject. We then rounded the result to nearest integer. If *s*_*ijk*_ = 0, we retained the original read count for the *i*^*th*^ gene, *j*^*th*^ group and *k*^*th*^ subject. In this way, *s*_*ijk*_ determine whether there exists an effect of the hidden variable on the *i*^*th*^ gene in the *j*^*th*^ group for the *k*^*th*^ subject. We repeated this whole process for *M* = 10 experiments.

After generating the count datasets, the set of differentially expressed genes were identified for each of the *M* experiments using edgeR in Bioconductor [14, 15] and the corresponding raw p-values of all the genes under study were stored. Then we applied our method EAMA to obtain the set of significant genes.

### Lung cancer datasets

We considered five publicly available lung cancer gene expression datasets: Bhattacharjee [16], GSE11969 [17], GSE29016 [18], GSE30219 [19] and GSE43580 [20]. These datasets were previously analyzed by [21] on a classification framework. Each of the datasets were normalized and filtered by [21]. All the five datasets were merged so that each of them had the same set of genes. We used the processed and merged datasets, which are available in the online Supporting informaton (S1 Datasets) as well as at https://zenodo.org/record/16006.

Each dataset had normalized expression levels for 7200 genes. Although, information regarding lung cancer type, smoking status, age and gender for the patients was available for our analysis, we only used the information about the cancer type of the patients.

## Results

### Simulated data

We called a gene differentially expressed if the corresponding “Benjamini-Hochberg” [6] adjusted p-value was less than 0.05. The performance of EAMA was assessed using four performance assessment measures: sensitivity, specificity, false discovery rate (FDR) and false non-discovery rate (FNR) as defined below:

1. Sensitivity (or true positive rate or recall): Proportion of genes that were correctly identified as differentially expressed among all the differentially expressed genes.
2. Specificity (or true negative rate): Proportion of genes that were correctly identified as non-differentially expressed among all the non-differentially expressed genes.
3. False discovery rate (FDR, or 1 minus precision): Proportion of genes that were incorrectly identified as differentially expressed among the set of identified differentially expressed genes.
4. False non-discovery rate (FNR): Proportion of genes that were incorrectly identified as non-differentially expressed among the set of identified non-differentially expressed genes.

The values of all the above four measures were calculated for EAMA based on 500 independent Monte-Carlo simulations. For comparison, we also obtained the results from a naïve meta-analysis which does not apply the empirical adjustment (null transformation) to the raw p-values. The naïve method combines the raw p-values using Fisher’s method [1] and adjust the resulting p-values using “Benjamini-Hochberg” method of multiplicity correction [6].

#### Continuous data (microarrays)

First, we considered the results from the scenario involving 10 independent experiments and 1000 uncorrelated genes where 70 genes were differentially expressed. The difference in magnitudes of the (log) expressions of these 70 genes between the two groups was 8.

**Fig 1:**
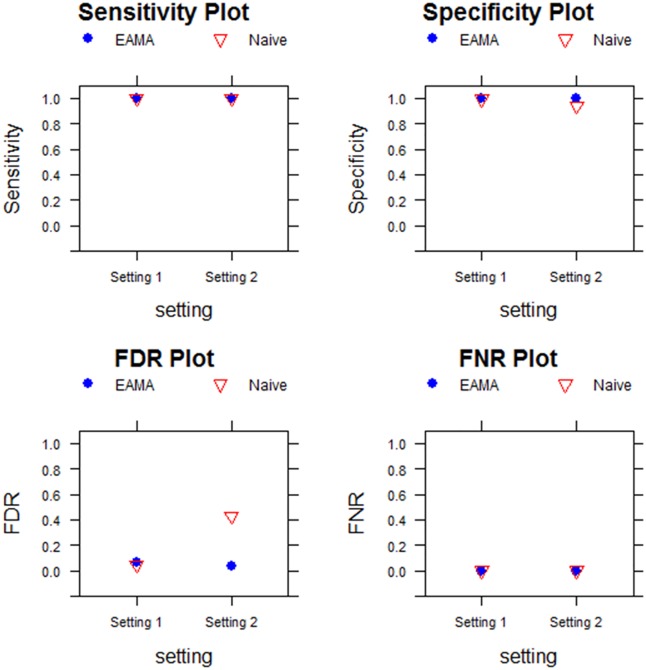
Performance assessment with 10 experiments, 1000 uncorrelated genes and absolute differences in differential expressions as 8.

Fig 1 shows the plots of sensitivity, specificity, FDR, and FNR of EAMA and that of the naïve method for each of the two simulation settings. From Fig 1, we found that the performances of EAMA and the naïve method were very similar in Setting 1 (no hidden confounder in the model) in terms of all the four performance measures. However, there was a wide difference between the performances of the two competing methods in Setting 2 (presence of hidden confounder in the model) as evident from Fig 1. The sensitivity of the EAMA was similar to that of naïve method, while the specificity of EAMA was much better than that of the naïve method. The most drastic difference was observed in the FDR measure in Setting 2. Here EAMA outperformed the naïve method by a large margin in terms of FDR, as the FDR of the naïve method was unacceptably high compared to that of EAMA.

From this simulation study, it appeared that the performances of EAMA and the naïve method were similar when there was no latent factor present in the study. But in the presence of some hidden variables which act as confounders and had significant effects on the expression levels of the genes, EAMA performed reasonably well in terms of having low FDR whereas the naïve method had unacceptably high FDR values. This result justifies our expectation that the EAMA, based on the empirical null distribution adjustment, lead to an accurate inference by accounting for the excess variations caused by the latent factors which were missed by the theoretical null based naïve method.

The performances of EAMA and that of the unadjusted (naïve) meta-analysis method were assessed using sensitivity, specificity, FDR, and FNR measures in each of the two simulation settings where the difference in magnitude of the (log) expression levels of the differentially expressed genes between the two groups was 8. Number of experiments involved in each setting was 10 and the number of genes (uncorrelated) considered was 1000.

#### i) Effect of reducing the difference in magnitude of the expression levels of the genes

**Fig 2:**
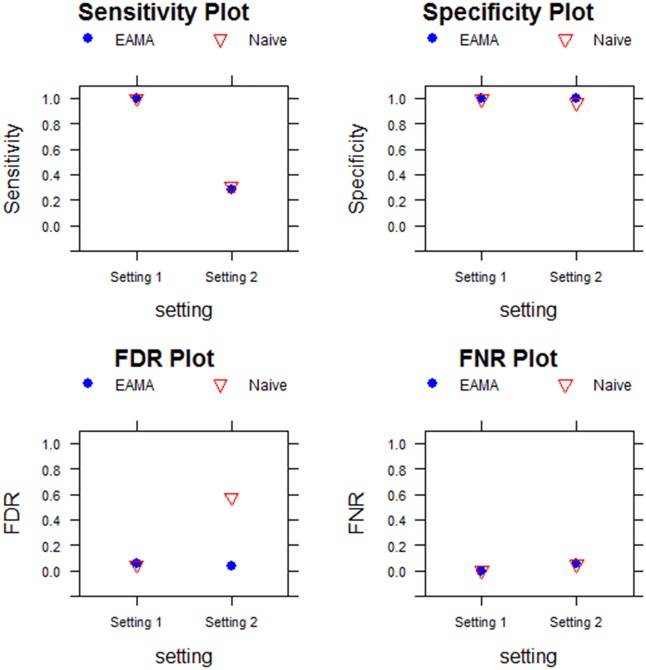
Performance assessment with 10 experiments, 1000 uncorrelated genes and absolute differences in differential expressions as 4.

In this case, too, we observed similar performances of EAMA and the naïve method in terms of sensitivity, specificity, and FNR in both the settings (see Fig 2). However, the difference between the FDR values of EAMA and the naïve method became higher with the performance of the naïve method deteriorating. As a result, the EAMA outperformed the naïve method by a very large margin. Interestingly, it appeared that the performance of the naïve method got worse as the difference in the magnitudes of differential expression tend to decrease.

The performances of EAMA and that of the unadjusted (naïve) meta-analysis method were assessed using sensitivity, specificity, FDR, and FNR measures in each of the two simulation settings where the difference in magnitude of the (log) expression levels of the differentially expressed genes between the two groups was 4. Number of experiments involved in each setting was 10 and the number of genes (uncorrelated) considered was 1000.

Since the most impactful difference between the performances of EAMA and the naïve method were obtained in terms of FDR in the presence of hidden variables acting as confounders, we have plotted the FDR values of both methods for a varying range of the differential effect (*δ*) of the confounder variable *W* in the two groups. S1 Fig in the online Supporting information section gives an idea of the patterns of FDR of the two methods for varying effects of the confounder.

#### ii) Effect of the presence of a hidden variable which does not act as a confounder (Setting 3)

We found that in Setting 3, where the latent variable no longer acts as a confounder, EAMA had higher sensitivity than the naïve method while the naïve method appeared a bit conservative with low sensitivity and FDR (see Table 1).

**Table 1:** Performance assessment of the two methods where a hidden variable does not act as a confounder.

Method	Sensitivity	Specificity	FDR	FNR
EAMA	0.517	0.995	0.0963	0.0351
Naive	0.337	0.999	0.023	0.0476

The online Supporting information section includes some additional simulation results corresponding to the cases of correlated genes, varying number of experiments, increased number of genes and varying effect of hidden variable or confounder. The results with correlated genes were found to be similar to what we obtained from the study of uncorrelated genes where EAMA performed much better than the naïve method in terms of FDR in the presence of latent factors in the study. See S2 Fig for details. Also, the relative performances of EAMA and the naïve method did not change with reduced number of experiments (*M* = 5), as seen in S3 Fig, and increased number of genes (see S4 Fig). For the simulation setting where the confounding effects exist in in individual experiments but tend to get nullified on combining the individual experiments, we see that EAMA continued to perform better than the naïve method in terms of having lower FDR (see S5 Fig).

#### Generation of count data (NGS data)

The performances of EAMA and that of the naïve meta-analysis method were assessed using the simulated count datasets as outlined in Experimental framework section. The results are shown in Table 2. From this table, we found that the EAMA and naïve method had similar performances in terms of sensitivity, specificity, and FNR, while the major difference lied in the FDR. The FDR of EAMA was much lower than that of the naïve method, and hence EAMA had less false discoveries. The results were similar to what we obtained in the studies involving continuous datasets.

**Table 2:** The performances of EAMA and that of the naïve method using the simulated count datasets.

Method	Sensitivity	Specificity	FDR	FNR
EAMA	0.870	0.968	0.129	0.032
Naïve	0.904	0.942	0.204	0.025

### Lung cancer data

We performed our meta-analysis based on the lung cancer datasets described under the Experimental framework section using the lung cancer type of the patients. Here, we attempted to identify the set of genes, which were differentially expressed between the two lung cancer types: Adenocarcinoma (AD) and Squamous cell carcinoma (SQ). The number of patients in each of the two types of lung cancer within each dataset is shown in Table 3.

**Table 3:** The number of patients in each of the two lung cancer types within each dataset.

Dataset	Lung cancer type	
	Adenocarcinoma (AD)	Squamous cell carcinoma(SQ)
Bhattacharjee	60	21
GSE11969	90	35
GSE29016	38	12
GSE30219	85	61
GSE43580	77	73

We fitted linear model with the gene expression values of the patients as the response and cancer type of the patients as the predictor, for each experiment separately. Using “limma” in Bioconductor [11] we obtained the p-values corresponding to the main effect term for the lung cancer type. So, we had five sets of p-values for each of the 7200 genes.

**Fig 3:**
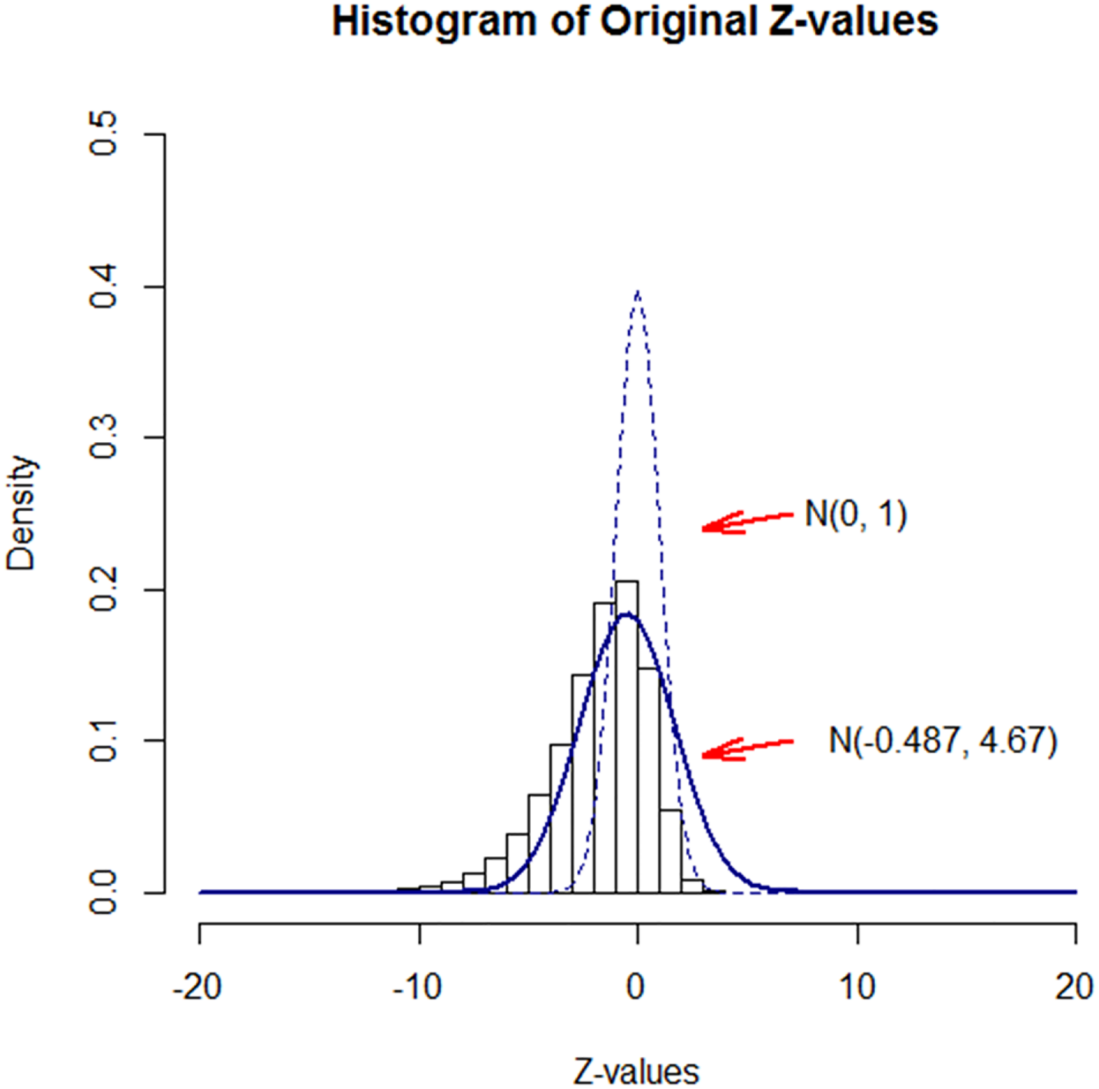
Histogram of the original z-values along with the empirical null distribution.

We identified the set of differentially expressed genes based on the five experiments using both EAMA and the naïve method. Fig 3 shows the histogram of the original z-scores obtained from Eq (2), while the two superimposed curves represent the empirical null distribution estimated from Efron’s method [2] and the theoretical null distribution. Note that the estimated mean and variance of the empirical null distribution turned out to be -0.487 and 4.67, respectively, which were much different from the theoretical null parameters of 0 (mean) and 1 (variance), respectively.

The naïve method identified 5127 differentially expressed genes (more than 70% of the total number of genes), even after adjusting for the “Benjamini-Hochberg” multiplicity correction method [6]. On the other hand, our proposed method EAMA identified 1541 significantly differentially expressed genes (approximately 21% of the total number of genes) after adjusting for “Benjamini-Hochberg” method of multiplicity correction [6], hence reducing the possibility of gross false discoveries.

**Fig 4:**
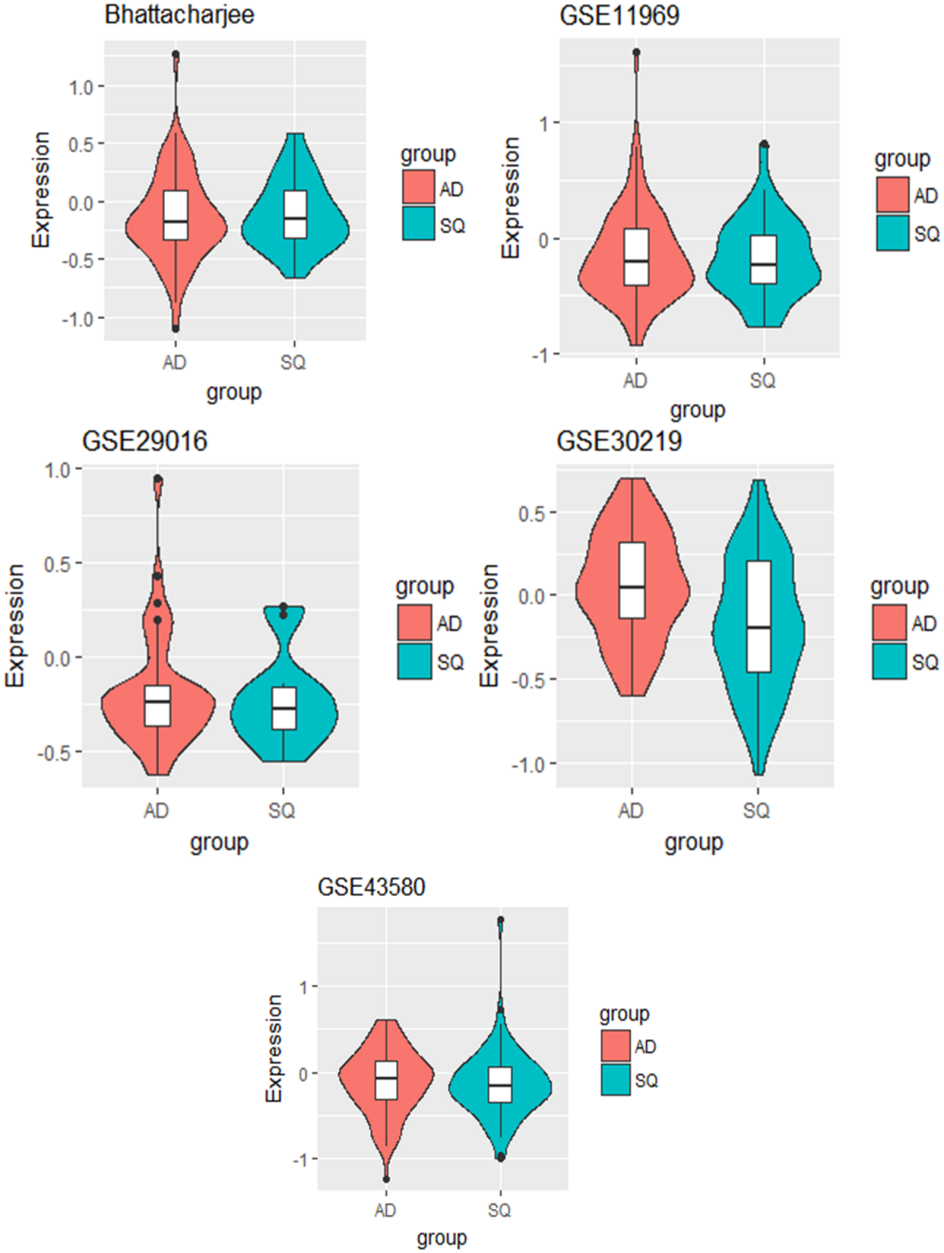
The violin plots of the gene with ID 472 for the two cancer types in each of the five datasets.

We further studied some of the genes that had been identified by the naïve method but not by EAMA. For example, the gene with ID 472 was identified by the naïve method but not by EAMA. We studied in details the expression pattern of this gene in each of the five datasets. Fig 4 shows the violin plots of the gene with ID 472 for the two cancer types in each of the five datasets.

From Fig 4, we find that the gene with ID 472 was not differentially expressed between the two lung cancer types in four of the five datasets. This suggested that the gene with ID 472, in spite of being identified by the naïve method, was unlikely to be an important factor for the discrimination between the two cancer types. Also, based on the individual analyses, the p-value corresponding to this gene was significant only for the dataset “GSE30219”.

**Fig 5:**
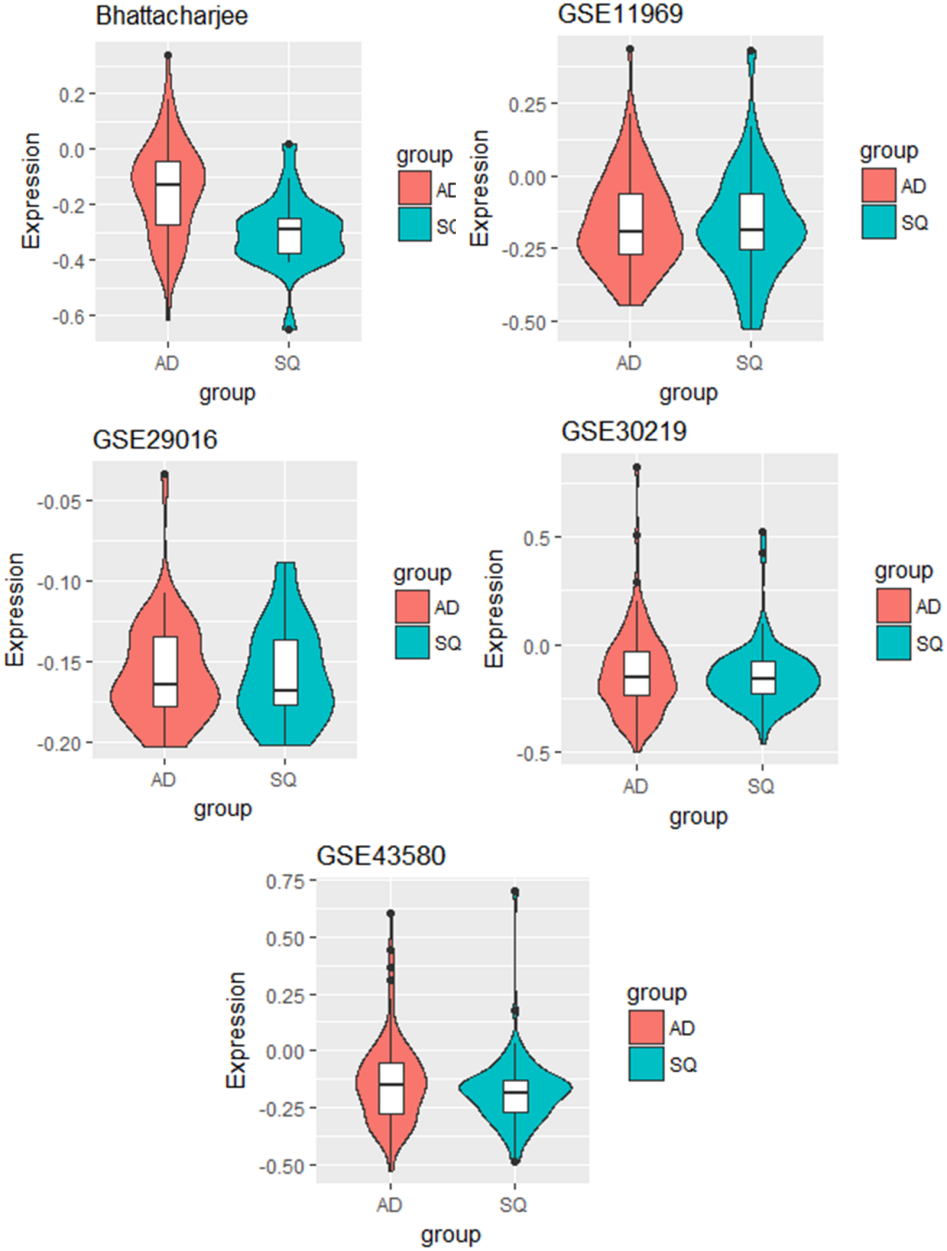
The violin plots for the gene with ID 8200 for the two cancer types in each of the five datasets.

Another such example is the gene with ID 8200 which was identified as significant by the naïve method but not by EAMA. Fig 5 shows the violin plots for the gene with ID 8200 for the two cancer types in each of the five datasets.

From Fig 5 we can see that the gene with ID 8200 was not differentially expressed between the two lung cancer types for four of the five datasets. As before, we can suggest that this gene did not have a considerable impact in distinguishing the two cancer types, and it was reasonable that EAMA had not identified such “unimportant” genes.

## Discussion

High throughput technologies have enabled simultaneous analysis of thousands of genes in a single experiment. Combining hypotheses testing results from the multiple genomic experiments is a popular meta-analysis approach of identifying significant genes related to some biological process. However, there is a distinct difference between the aims of meta-analyses involving single hypothesis in each component experiment with that of large-scale multiple hypotheses in each experiment. While the former targets in favoring the alternative ‘interesting’ hypothesis with high power, the latter is designed for the identification of a small proportion of ‘interesting’ or ‘significant’ cases out of a large set of possible candidates. In this article we have discussed the possibility of making erroneous conclusions from combining the p-values calculated under standard theoretical assumptions from multiple genomic experiments when each experiment involves simultaneous testing of enormous number of hypotheses in presence of some hidden confounder variable. The presence of some confounder variables can induce over-dispersion and/or bias that remain unaccounted by the theoretical null assumptions. In particular, we have shown that even adjusting the p-values by taking into account the false discovery rates may not be enough to substantially diminish the false discoveries from the meta-analysis of large-scale multiple testing when hidden confounder variables are present. We have proposed an alternative approach of modifying the p-values by constructing empirical null distributions and combining these empirically adjusted p-values through proper meta-analysis approach. Through simulation studies involving genomic experiments we have shown that our proposed method has much better performance than the standard meta-analysis approach of combining raw p-values from multiple experiments especially in presence of hidden confounder variables. A possible example where the Fisher’s p-value combination method may lead to incorrect findings can be found from our real data analysis. In our meta-analysis of five lung cancer data sets, we have discussed examples where certain genes were identified as differentially expressed by the standard method of Fisher’s p-value combination, although individual analyses of the five lung cancer datasets reveal that the aforementioned genes were not differentially expressed in most of the five component datasets. This strongly raises the possibility of false discoveries using Fisher’s p-value combination due to some unmeasured confounder in at least one of those datasets.

There have been some proposed model-based methods for gene-expression studies that take into account the potential effects of confounders while carrying out genomic analyses. One such model based approach, which is applicable for individual genomic studies, is the surrogate variable analysis [22]. Such model-based approaches to account for the effects of the confounders have also been applied for meta-analysis purposes [3]. However, in practice, it may become difficult and complicated to model the effects of hidden confounders in large observational studies due to various complex or unknown nature of the confounding effects. In such situations, it might be more useful to empirically adjust for the effects of hidden confounders, through our proposed method, instead of going through cumbersome modeling approaches.

In genome wide association studies (GWAS) there exist some other popular approaches of meta-analysis apart from the Fisher’s method of p-value combination. One such popular meta-analysis method is the METAL approach [23] which combines the p-values or effect sizes of individual association studies by weighted combination and transformation into z-values where the weights depend on either the sample sizes or the estimated standard errors of the effect sizes of the individual studies. This approach, although useful for meta-analysis of GWAS, does not appropriately account for the effects of the potential confounders which are likely to be present in large-scale observational studies. In this article, we have developed our method of empirically adjusted meta-analysis based on the Fisher’s p-value combination due to the flexibility and computational ease of the Fisher’s method. However, in case additional information on effect sizes and corresponding standard errors are available for the individual component studies, one can also implement our technique of empirical adjustment to METAL for further improved meta-analysis results.

This article mainly focuses on developing meta-analysis approach for combining p-values from multiple genomic experiments when the outcome of interest is affected by some hidden variable that acts as a confounder with different effects between different groups under study. In biological studies, mostly in observational studies, such hidden variables are very common which play the roles of confounders. We have shown that in such scenarios our proposed method performs much better than the standard naïve method of meta-analysis. However, it may happen in certain situations that a hidden variable affects the outcome but does not have a confounding effect, i.e. the effect of the hidden variable on the outcome does not vary significantly between the different groups under study. We have considered multiple simulation settings that addresses the abovementioned scenarios and the results show that the standard meta-analysis approach of combining raw p-values is a bit conservative having lower power and FDR than our proposed method EAMA.

We have used empirical null distribution as outlined by [2] where the empirical null distribution were estimated using a “central matching” approach (see Appendix as well as [24] for details) of the R package “locfdr” [25]. There is an alternative option of estimating the null distribution using the maximum likelihood method in the “locfdr” package (Details can be found in [24]). In various simulation scenarios, the “central matching” approach of estimating the empirical null distribution appeared to perform better than the maximum likelihood approach. However, the overall results obtained through the maximum likelihood approach are not significantly different from that obtained from the “central matching” approach and are not shown here in details. One may note that although we have used FDR-adjusted p-values using Benjamini-Hochberg procedure [6], there are other options for adjustment of false discovery rates including the use of q-values [26].

## Methods

### Empirically adjusted meta-analysis (EAMA)

Here, we provide a description of our proposed method (EAMA) in details. Let us suppose we have *G* number of genes in our study. We are interested in finding out which of these *G* genes contribute significantly to our outcome of interest. Suppose *H* = {*H*_*g*_:1 ≤ *g* ≤ *G*} be the collection of null hypotheses where *H*_*g*_ denotes the hypothesis that the gene *g* has no significant contribution to the outcome of interest, 1 ≤ *g* ≤ *G*. Also, suppose we have data from multiple independent experiments. Let *M* be the number of independent genomic experiments. By combining the results of these *M* independent experiments we aim to identify the genes which contribute significantly to the outcome of interest.

Let us define $P^{\left(i\right)}=\left\{{\hat{p}}_{g}^{\left(i\right)}:1\leq g\leq G\right\}$ as the collection of p-values from the *i*^*th*^ genomic experiment, where ${\hat{p}}_{g}^{\left(i\right)}$ is the p-value corresponding to gene *g* (i.e. corresponding to the null hypothesis *H*_*g*_) in the *i*^*th*^ experiment, 1 ≤ *i* ≤ *M*, 1 ≤ *g* ≤ *G*.

Using the inverse z-transformation, we get the collection of z-scores as

$$z^{\left(i\right)}=\left\{z_{g}^{\left(i\right)}=\Phi^{-1}({\hat{p}}_{g}^{\left(i\right)}):1\leq g\leq G\right\},\;1\leq i\leq M$$ (2)

The z-scores given in (2) may not follow a *N*(0, 1) distribution under the null hypotheses. Here we modify the z-scores, given in (2), using the Efron’s technique of estimating an empirical null distribution [2] so that the resulting z-scores follow *N*(0, 1) distribution under the null hypotheses. A brief detail on estimating the empirical null can be found in the Appendix. Following the steps of [2], suppose the empirical null distribution is obtained as $\;{\hat{f}}_{0}\;=\;N({\hat{\mu }}_{0},{{\hat{\sigma }}_{0}}^{2})$ using the R package “locfdr” [25]. Then the modified z-values are calculated as

$${\tilde{z}}_{g}^{\left(i\right)}=\frac{z_{g}^{\left(i\right)}-{\hat{\mu }}_{0}}{{\hat{\sigma }}_{0}},1\leq g\leq G,\;1\leq i\leq M$$ (3)

These empirically adjusted z-values, given in (3), can be assumed to follow a *N*(0, 1) distribution under the appropriate null hypotheses. Finally, we convert the modified z-values into the corresponding p-values as $\;{\tilde{p}}_{g}^{\left(i\right)}=\Phi ({\tilde{z}}_{g}^{\left(i\right)}),\;1\leq g\leq G,\;1\leq i\leq M$.

At this stage, we have a set of *M* p-values from the *M* independent experiments for each gene *g*. But for proper inference on the overall effect of a gene, we need to have a single p-value for that gene. So, for a typical gene *g* we combine the p-values from all of the *M* experiments to obtain a single p-value using Fisher’s method [1] as given below:

If ${\;\tilde{p}}_{g}^{\left(1\right)},\;{\tilde{p}}_{g}^{\left(2\right)},\ldots ,\;{\tilde{p}}_{g}^{\left(M\right)}\;$ are the *M* p-values for the gene *g* obtained from the *M* independent experiments, we combine these *M* p-values to get a single test statistic $\;{\;T}_{g}=2\sum_{i\;=\;1}^{M}\left\{-log({\tilde{p}}_{g}^{\left(i\right)})\right\}$, 1 ≤ *g* ≤ *G*. Under the null hypothesis *H*_*g*_: gene *g* does not contribute significantly to the outcome, *T*_*g*_ follows a *χ*^2^ distribution with 2*M* degrees of freedom assuming that the p-values ${\;\tilde{p}}_{g}^{\left(i\right)}$ follow uniform distribution. Using these we obtain a resulting set of *G* p-values $\left\{{\tilde{p}}_{1},\;{\tilde{p}}_{2},\;\ldots ,\;{\tilde{p}}_{G}\right\}$, where ${\tilde{p}}_{g}$ is the p-value corresponding to the test statistic *T*_*g*_. In this way we are able to get a single p-value corresponding to each of the *G* genes. To account for the large number of hypotheses being tested in the study, we apply the Benjamini-Hochberg [6] method of multiplicity correction to get a set of corrected p-values $\left\{p_{1}^{*},\;p_{2}^{*},\;\ldots ,\;p_{G}^{*}\right\}$. It may be noted that other methods for multiplicity correction can also be used. Finally, using the p-value $p_{g}^{*}$ we decide whether the gene *g* has any overall significant contribution to the outcome of interest.

## Appendix

### Estimation of the empirical null distribution

A brief discussion on estimating the null distribution, following [2], is as follows. Suppose that there are *N z* -values which can be classified into two classes, “Uninteresting” or “Interesting”, depending on whether or not *z*_*i*_ is generated according to the null hypothesis. Also, assume that the prior probabilities of the *z*-values belonging to the “Uninteresting” or “Interesting” classes are *p*_0_ and *p*_1_ = 1 − *p*_0_ respectively, and that *z*_*i*_ has density either *f*_0_(*z*) (null density) or *f*_1_(*z*) (non-null) depending on its class. Then the mixture density of *z* is given by *f*(*z*) = *p*_0_*f*_0_(*z*) + *p*_1_*f*_1_(*z*). Following Bayes theorem the *a posteriori* probability of belonging to the Uninteresting class given *z* is obtained as Prob{Uninteresting|*z*} = *p*_0_*f*_0_(*z*)/*f*(*z*). The local false discovery rate is then defined as *f*_0_(*z*)/*f*(*z*). The main idea is to estimate the density *f*_0_ from the central peak of the observed histogram of the *z*-values. Under the assumption that *f*_0_ is density of a normal distribution with mean *δ*_0_ (not necessarily 0) and standard deviation *σ*_0_ (not necessarily 1), for *z* close to zero, we can write $\;logf(z)=-\frac{1}{2}{\left(\frac{z-\delta_{0}}{\sigma_{0}}\right)}^{2}+constant$. Then *δ*_0_ and *σ*_0_ can be estimated as *δ*_0_ = *argmax*{*f*(*z*)} and ${\;\sigma }_{0}\;=\;{\left[-\frac{d^{2}}{dz^{2}}logf(z)\right]}_{\delta_{0}}^{-\frac{1}{2}}$. Around *z* = 0, *logf*(*z*) curve is estimated through a quadratic approximation which leads to the final estimation of *δ*_0_ and *σ*_0_. This is done under the assumption that the central peak of the *z*-value histogram, presumably close to zero, is mainly contributed by the null cases, and this method of estimating the null parameters is termed as “central matching” method (see [24] for more details on this approach).

## Supporting information

S1 Text Details of the additional analyses. (DOCX)

S1 Fig Comparison of FDR values of EAMA and the naïve method. The FDR values of both EAMA and the naïve method were compared for a varying range of the differential effect of the hidden confounder variable in the two groups. Number of genes considered was 100000 and the number of experiments considered was 10. The difference in magnitude of the (log) expression levels of the differentially expressed genes between the two groups was 8.
(DOCX)

S2 Fig Performances of EAMA and the naïve method with correlated genes. The performances of EAMA and that of the naïve meta-analysis method were assessed using sensitivity, specificity, FDR, and FNR measures in each of the two simulation settings where the difference in magnitude of the (log) expression levels of the differentially expressed genes between the two groups was 8. Number of experiments involved in each setting was 10 and the number of genes (correlated) considered was 1000.
(DOCX)

S3 Fig Effect of reducing the number of experiments on the performances of EAMA and naïve method. The performances of EAMA and that of the naïve meta-analysis method were assessed using sensitivity, specificity, FDR, and FNR measures in each of the two simulation settings with reduced number of experiments. The difference in magnitude of the (log) expression levels of the differentially expressed genes between the two groups was 8. Number of genes (uncorrelated) considered was 1000 and the number of experiments considered was 5.
(DOCX)

S4 Fig Effect of increasing the number of genes on the performances of EAMA and naïve method. The performances of EAMA and that of the naïve meta-analysis method were assessed using sensitivity, specificity, FDR, and FNR measures in each of the two simulation settings with increased number of genes. The difference in magnitude of the (log) expression levels of the differentially expressed genes between the two groups was 8. Number of genes (uncorrelated) considered was 100000 and the number of experiments considered was 10.
(DOCX)

S5 Fig Nullified effect of confounders on combining multiple experiments. The performances of EAMA and that of the naïve meta-analysis method were assessed in each of the two simulation settings. In Setting 2 of this scenario the confounder affects two of the component experiments, but, in such a way that the confounding effect in one experiment tends to cancel the other. The difference in magnitude of the (log) expression levels of the differentially expressed genes between the two groups was 8. Number of genes (uncorrelated) considered was 1000 and the total number of experiments considered was 10.
(DOCX)

S1 Datasets Datasets used for analysis. (ZIP)
