# Supplementary material for: EAMA: Empirically adjusted meta-analysis for large-scale simultaneous hypothesis testing in genomic experiments
Source: PLoS One. 2017 Oct 31;12(10):e0187287. doi: 10.1371/journal.pone.0187287 (PMC5663489; doi:10.1371/journal.pone.0187287)
Supplement: S1 Text — (DOCX) [file pone.0187287.s001.docx]

**Comparison of FDR values for EAMA and the naïve method varying the effect of the hidden confounder**

We also compared the FDR values of EAMA with that of the naïve method over a range of magnitude of the effect of the hidden confounder,$W$. Here, we generated $W_{ijk}$ through the generation of$u_{ijk}$ as before in Setting 2. In order to vary the magnitude of the effect of the hidden confounder, we vary the magnitude of the difference $\left( \delta\right)$ between the means of the distributions of $u_{ijk}$ in the two groups as given below:

$$u_{i1k} \sim\left\{ \begin{matrix} N\left( -1+m, {0.01}^{2} \right) & \mathrm{for} 1\leq i\leq2000; k=1,2,\ldots,10 \\ N\left( 2+m, {0.01}^{2} \right) & \mathrm{for} 2001\leq i\leq8000; k=1,2,\ldots,10 \\ N\left( 5+m, {0.01}^{2} \right) & \mathrm{for} 8001\leq i\leq100000; k=1,2,\ldots,10 \end{matrix} \right.$$

and

$$u_{i2k} \sim\left\{ \begin{matrix} N\left( -1+\delta+m, {0.01}^{2} \right) & \mathrm{for} 1\leq i\leq2000; k=11,12,\ldots,20 \\ N\left( 2+\delta+m, {0.01}^{2} \right) & \mathrm{for} 2001\leq i\leq8000; k=11,12,\ldots,20 \\ N\left( 5+\delta+m, {0.01}^{2} \right) & \mathrm{for} 8001\leq i\leq100000; k=11,12,\ldots,20 \end{matrix} \right.$$

where $m$ denotes the experiment ID. We considered different choices of $\delta$ as 1, 1.5, 2, 2.5, 3, 3.5, and 4. After generating the microarray datasets, we applied EAMA as well as the naïve method to obtain the set of significant genes.

**Correlated gene expression levels**

Genes having same biological functions are expected to have correlated expression values in the datasets. To study the robustness of EAMA, we generated a simulation scenario where there were 4 clusters of correlated genes. We used the same model (1) from the Experimental Framework of the main article in generating the (log) expression values, similar to the scenario of independent genes, but assured correlation among the genes within a cluster through the generation of the random error term $\left( e_{ijk} \right)$ in the following way:

Let $C_{1}=\left\{ 1, 2,\ldots, 10 \right\}, C_{2}=\left\{ 41, 42,\ldots, 60 \right\}, C_{3}=\left\{ 501, 502,\ldots, 520 \right\}$ and $C_{4}=\left\{ 891, 892,\ldots, 910 \right\}$ denote the 4 clusters and $C=\left\{ C_{1}, C_{2}, C_{3}, C_{4} \right\}$ denotes the union of the 4 clusters.

We generated $e_{ijk}$as

$e_{ijk}=\left\{ \begin{matrix} \frac{1}{\sqrt{2}}e_{ijk}^{1}+\frac{1}{\sqrt{2}}e_{ijk}^{2} & if i \epsilon C \\ e_{ijk}^{2} & o.w \end{matrix} \right.$, $i=1,2,\ldots, G;j=1,2;k=1,2,\ldots, N$

where $e^{1}$ were drawn independently from $N\left( 0, 1 \right)$ in such a way that the values of $e^{1}$were same for all the genes belonging to the same cluster. $e^{2}$ were generated independently from $N\left( 0, 2^{2} \right)$. All the other variables in (1) were generated in two different ways, similar to the studies with independent genes, namely Setting 1 and Setting 2. After generating the microarray datasets with correlated gene expression values, we applied EAMA and the naïve method to obtain the set of significant genes.

**Reduction of the number of experiments**

We considered simulation scenarios with reduced number of experiments. In particular, we considered the number of experiments$(M)$ to be 5. The (log) expression values of the genes were generated using (1) in the same way as described in the Experimental Framework of the main article, i.e. using (1), and under the two settings, namely, Setting 1 and Setting 2. After generating the microarray datasets with reduced number of experiments, we applied both EAMA and the naïve method to obtain the set of significant genes.

**Increase in the number of genes**

We considered simulation scenarios where the number of genes (hypotheses) involved was increased to 100,000. The (log) expression values of the genes were generated in the same way as previous scenarios, i.e. using (1), and under the two settings, namely, Setting 1 and Setting 2. We applied both EAMA and the naïve method to obtain the set of significant genes in this scenario.

**Nullified effect of confounders on combining multiple experiments**

We considered a simulation scenario, a variation of setting 2, where the confounder affects some of the component experiments, but, in such a way that the overall effect of the confounder gets nullified on combining the individual studies. In particular, the gene expression values were generated through (1) as described in the Experimental Framework of the main article with the choice of the number of experiments$(M)$ as 10, such that the hidden variable$W_{ijk}$ was 0 for all values of $m$ (experiment ID) other than 9 and 10. For $m=9, 10$, we generated $W_{ijk}$ as follows.

$W_{ijk}=u_{ijk}I\left( s_{ijk}=1 \right)$, where$s_{ijk} \sim\mathrm{Bernoulli}\left( 0.4 \right)$ and

$$u_{i1k} \sim\left\{ \begin{matrix} N\left( -1+\left( -1 \right)^{m}\times10, {0.01}^{2} \right) & \mathrm{for} 1\leq i\leq20; k=1,2,\ldots,10 \\ N\left( 2+\left( -1 \right)^{m}\times10, {0.01}^{2} \right) & \mathrm{for} 21\leq i\leq70; k=1,2,\ldots,10 \\ N\left( 5+\left( -1 \right)^{m}\times10, {0.01}^{2} \right) & \mathrm{for} 71\leq i\leq1000; k=1,2,\ldots,10 \end{matrix} \right.$$

and

$$u_{i2k} \sim\left\{ \begin{matrix} N\left( -1+\delta+\left( -1 \right)^{m}\times10, {0.01}^{2} \right) & \mathrm{for} 1\leq i\leq20; k=11,12,\ldots,20 \\ N\left( 2+\delta+\left( -1 \right)^{m}\times10, {0.01}^{2} \right) & \mathrm{for} 21\leq i\leq70; k=11,12,\ldots,20 \\ N\left( 5+\delta+\left( -1 \right)^{m}\times10, {0.01}^{2} \right) & \mathrm{for} 71\leq i\leq1000; k=11,12,\ldots,20 \end{matrix} \right.$$

Here,$\delta=4$, and the random errors $\left( e_{ijk} \right)$ in model (1) are generated from $N\left( 0, {0.8}^{2} \right)$. In this scenario the hidden confounder affects only two of the ten experiments, namely, $m=9, 10$, but the confounding effect in one experiment tends to cancel the other. We applied both EAMA and the naïve method to obtain the set of significant genes in this scenario.
