# Supplementary figures and images for: EAMA: Empirically adjusted meta-analysis for large-scale simultaneous hypothesis testing in genomic experiments
Source: PLoS One. 2017 Oct 31;12(10):e0187287. doi: 10.1371/journal.pone.0187287 (PMC5663489; doi:10.1371/journal.pone.0187287)

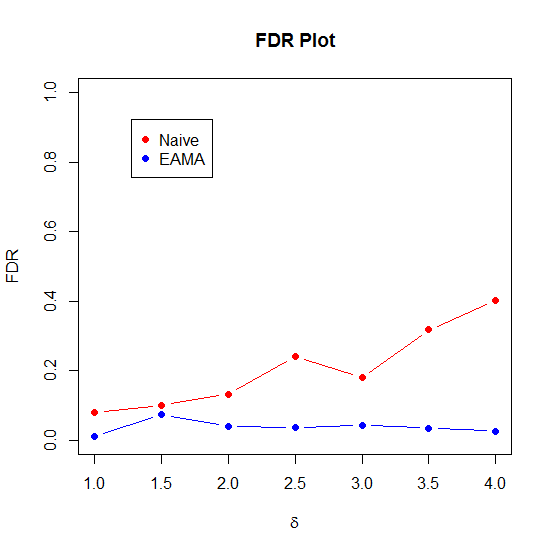

Supplement: S1 Fig — The FDR values of both EAMA and the naïve method were compared for a varying range of the differential effect of the hidden confounder variable in the two groups. Number of genes considered was 100000 and the number of experiments considered was 10. The difference in magnitude of the (log) expression levels of the differentially expressed genes between the two groups was 8. (DOCX) [file pone.0187287.s002.docx]

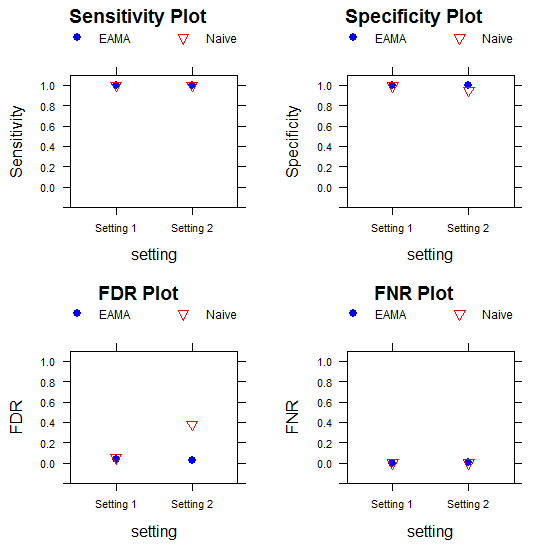

Supplement: S2 Fig — The performances of EAMA and that of the naïve meta-analysis method were assessed using sensitivity, specificity, FDR, and FNR measures in each of the two simulation settings where the difference in magnitude of the (log) expression levels of the differentially expressed genes between the two groups was 8. Number of experiments involved in each setting was 10 and the number of genes (correlated) considered was 1000. (DOCX) [file pone.0187287.s003.docx]

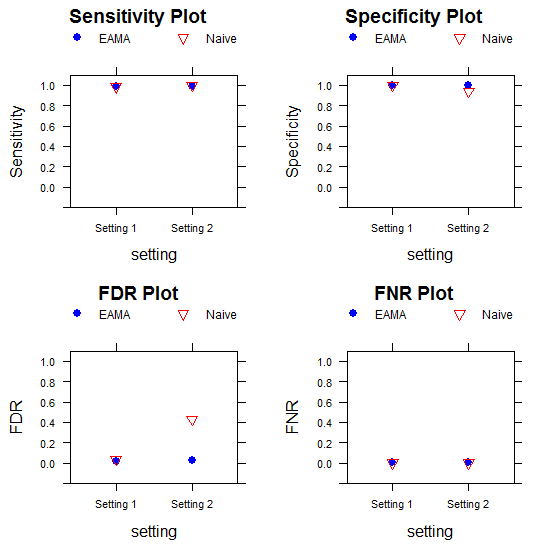

Supplement: S3 Fig — The performances of EAMA and that of the naïve meta-analysis method were assessed using sensitivity, specificity, FDR, and FNR measures in each of the two simulation settings with reduced number of experiments. The difference in magnitude of the (log) expression levels of the differentially expressed genes between the two groups was 8. Number of genes (uncorrelated) considered was 1000 and the number of experiments considered was 5. (DOCX) [file pone.0187287.s004.docx]

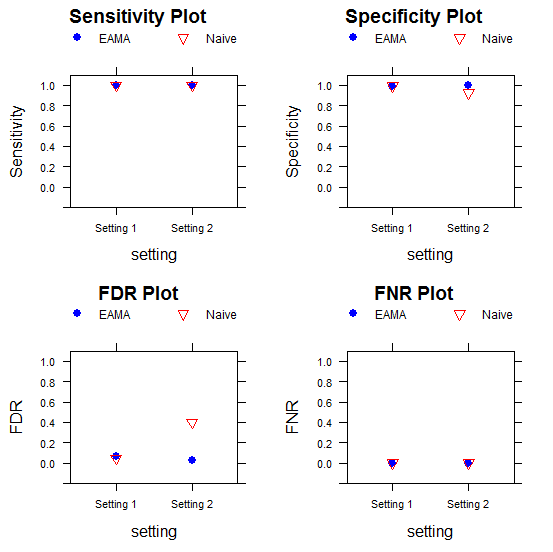

Supplement: S4 Fig — The performances of EAMA and that of the naïve meta-analysis method were assessed using sensitivity, specificity, FDR, and FNR measures in each of the two simulation settings with increased number of genes. The difference in magnitude of the (log) expression levels of the differentially expressed genes between the two groups was 8. Number of genes (uncorrelated) considered was 100000 and the number of experiments considered was 10. (DOCX) [file pone.0187287.s005.docx]

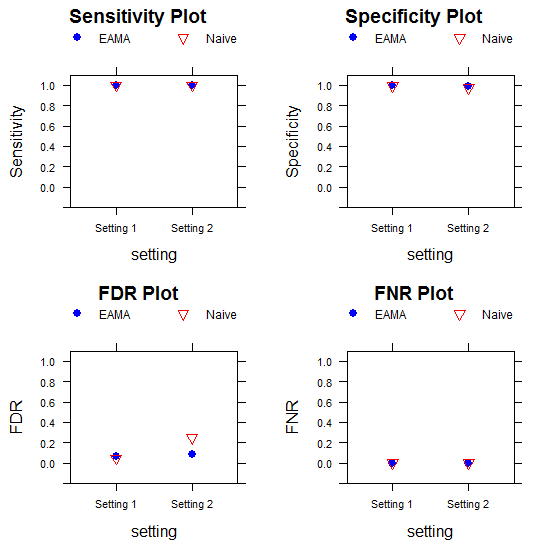

Supplement: S5 Fig — The performances of EAMA and that of the naïve meta-analysis method were assessed in each of the two simulation settings. In Setting 2 of this scenario the confounder affects two of the component experiments, but, in such a way that the confounding effect in one experiment tends to cancel the other. The difference in magnitude of the (log) expression levels of the differentially expressed genes between the two groups was 8. Number of genes (uncorrelated) considered was 1000 and the total number of experiments considered was 10. (DOCX) [file pone.0187287.s006.docx]
